# The impact of fine particulate matter on depression: Evidence from social media in China

**DOI:** 10.1371/journal.pone.0320084

**Published:** 2025-03-31

**Authors:** Yao Zhong, Jianxin Guo, Hongbiao Wang, Zhufeng Qiao, Jichun Zhao, Lei Chen, Ying Nie

**Affiliations:** Institute of Data Science and Agricultural Economics, Beijing Academy of Agriculture and Forestry Sciences, Beijing, China; The Fourth People’s Hospital of Chengdu, CHINA

## Abstract

Depression is a significant public health issue in China that imposes a heavy economic burden on society and families. Using a dataset of 8.54 million Weibo posts from 284 prefecture-level cities across China between 2016 and 2019, we calculate the depression tendency index for residents in each city. Using the weighting of pollutants in nearby cities as an instrumental variable, we apply the two-stage least squares method to estimate the impact of PM2.5 on depression. The findings reveal that (1) air pollution markedly influences residents’ susceptibility to depression, and every 1 μg/m^3^ increase in the PM2.5 concentration results in a 0.0559% increase in the depression tendency value. (2) The influence of air pollution on residents’ depression exhibits a distinct weekly pattern, with individuals in heating cities, on weekdays, and in lower-income brackets being more impacted. (3) Our analysis of healthcare expenditures affirms that China’s environmental governance policies have yielded significant economic advantages. As mitigation strategies, we propose the adoption of air pollution evasion measures, persistent refinement and enforcement of air pollution regulatory policies to reduce environmental pollution-related damage, paying attention to groups at risk of depression and fostering a healthy society.

## 1. Introduction

Recent studies have confirmed that air pollution causes respiratory and circulatory diseases and significantly increases mortality rates from heart and lung diseases, including lung cancer [1–3]. Air pollution can also have negative effects on mental health, including depression and cognitive impairment, leading to reduced subjective well-being and enormous economic losses. Compared with physiological diseases, the stigmatization of mental illnesses by society leads to lower rates of seeking medical treatment for mental illnesses [4,5]. Therefore, there is a possibility of underestimating the psychological and socioeconomic costs caused by air pollution. A comprehensive and sustained assessment is crucial to making environmental policy decisions, and cost-benefit trade-offs and implementation intensity choices must be considered.

Air pollution has always been the primary environmental problem in China [6,7]. Although China has strengthened air quality monitoring and governance since 2016 [8], 53.4% of cities in China still did not meet national air quality standards in 2019 [9]. Air pollution continues to pose a health threat, particularly with depression related to mental health, which is receiving increasing attention [10]. Medical and psychology research has provided some indirect evidence. Medical studies have confirmed that the neurotoxicity of air pollutants can lead to inflammation and oxidation in the prefrontal cortex, resulting in cerebrovascular damage and neurodegenerative diseases that increase the risk of depression [11] and even suicide due to depression [12]. According to environmental psychology, exposure to air pollution exacerbates individual stress responses and damages mental health [13,14]. Additionally, air pollution limits outdoor activities and social interactions, increasing negative emotions [15,16].

Although there is some evidence of the negative impact of short-term air pollution on mental health in countries in Europe, emergency data confirm the positive correlation between various air pollutants and the number of emergency visits for depression [17,18]. However, research on the effects of medium and long-term exposure is still insufficient. Research has preliminarily confirmed a significant correlation between air pollution and depression using annual PM2.5 data, but the author also noted that the use of cross-sectional data may lead to a reverse causal relationship [19]. In addition, researchers often use happiness or negative emotions as indicators to explore the impact of air pollution on mental health, with limited direct assessment of depression [20,21]. In China, there is even less direct and effective research on depression caused by air pollution, especially with respect to short-term stress damage and medium- and long-term cumulative effects.

One of the important reasons is the availability of data. Diagnostic data or epidemiological survey data from large sample sizes are often scarce. The existing studies on depression have relied mainly on questionnaire survey data, but self-reported data are prone to bias which can reduce the accuracy of the results. Furthermore, medical resources are typically used to control and intervene in the occurrence and treatment of infectious and physiological diseases [22], which may result in underestimating the number of patients with depression. Currently, depression has become one of the top three sources of disease burden in China, and the number of patients with depression is on the rise, from 54 million in 2017 to 95 million in 2019, with a growth rate of 76% [23]. Thus, it is imperative to expand other data sources and research methods. Social media big data provides promising possibilities in this regard [24,25].

An increasing number of people use Twitter, Weibo, and other social media platforms to express their opinions and emotions. As a result, social media discourse has become a valuable source of data for research in fields such as medicine, mental health, and behavioral economics [26]. Social media data are characterized by a large data volume and wide geographical distribution, and are naturally generated in a noninvasive manner, reducing the difficulty and cost of data collection [27,28]. The commonly used method is natural language processing technology, which can automatically process and analyze large amounts of text data, mine text emotions, effectively identify the characteristics of depression patients, and effectively improve data processing efficiency and accuracy [29,30]. Similar methods have also been applied in social media research in China, with some studies using natural language processing to construct a daily happiness index for cities based on Weibo text data [20], or with the help of an open Chinese sentiment analysis platform to classify positive and negative emotions [24]. However, there is relatively little research on subdividing depressive emotions in terms of mental health. Research on Chinese social media big data faces challenges due to language differences. The classification of depression in Chinese online texts cannot rely on existing English natural language processing tools. Therefore, according to related research on Chinese social media big data, specific research on depression or mental health is not feasible when these methods are used alone.

This study focuses on Chinese social media sentiment analysis of the specific mental health branch of depression, which provides more direct evidence for health damage assessment of air pollution. The preliminary hypothesis underlying our research is that keeping other socioeconomic and environmental variables constant, an increase in the PM2.5 concentration significantly promotes depression among urban residents. In terms of sample selection, as Weibo is currently the largest open social media platform in China, users can quickly and real-time share information about their lives. In 2019, there were 516 million monthly active users and 222 million daily active users, and the monthly active users have covered 60.42% of China’s 854 million netizens, widely distributed in first, second, third, and fourth tier cities and covering various age groups [31–33]. Weibo users are mainly composed of middle-aged and young people, with a coverage ratio similar to that of Chinese netizens. Existing research explores the impact of air pollution on sleeplessness, tourists’ emotions, and individual emotions based on Weibo text data [34–36]. Weibo users highly represent Chinese netizens. Although netizens cannot represent all residents, they do represent a portion of residents. Therefore, based on data availability, we applied 8.54 million Weibo texts between January 1, 2016, and December 31, 2019, and constructed a daily Weibo depression propensity index for 284 prefecture-level cities via natural language processing technology. To overcome the endogenous influence caused by missing variables, we conducted a two-stage least squares estimation (2SLS) using the air pollution spillover effect in the upwind area as the instrumental variable. This study aims to provide empirical evidence for the short-term and mid-term cumulative impact of air pollution on mental health and provides a reference for objectively and comprehensively assessing social welfare losses caused by air pollution. The study is also a decision-making reference for environmental policy optimization and cost-benefit measurement.

## 2. Materials and methods

### 2.1. Sample and data

Numerous research employed by using Python crawlers or Weibo’s public application programming interface (API) (https://open.weibo.com/wiki/%E9%A6%96%E9%A1%B5) to obtain Weibo text data [37,38]. This study refers to previous literature and uses web crawler technology to obtain Weibo text data using keywords such as ‘depressed’, ‘negative’, and ‘lethargic (Mei Jing shen)’, and obtains Weibo user information through the open API of Sina Weibo. The Weibo text information includes the user ID, Weibo text ID, Weibo text content, publication time, etc. The Weibo user information includes the user ID, province, city, number of followers, etc. Weibo text information and user information can be matched through user IDs.

In terms of time range selection, as the internet penetration rate reached 50% in 2016, the number of active users on Weibo passed 40% of Chinese netizens, and the proportion of internet users in China reached 60% in 2019 [39]. Weibo users gradually expanded from first- and second-tier cities such as Beijing and Hangzhou to third- and fourth-tier cities and below such as Yinchuan and Yushu in 2016. Therefore, the number of Weibo users in third- and fourth-tier cities in the previous two years was relatively small [40]. Additionally, starting on January 1, 2016, the release of local air quality prediction and forecast information was obligatory for prefecture level cities in China [8]. Considering the completeness of sample acquisition, daily data for four years from January 1, 2016, to December 31, 2019, were selected.

**Fig 1:**
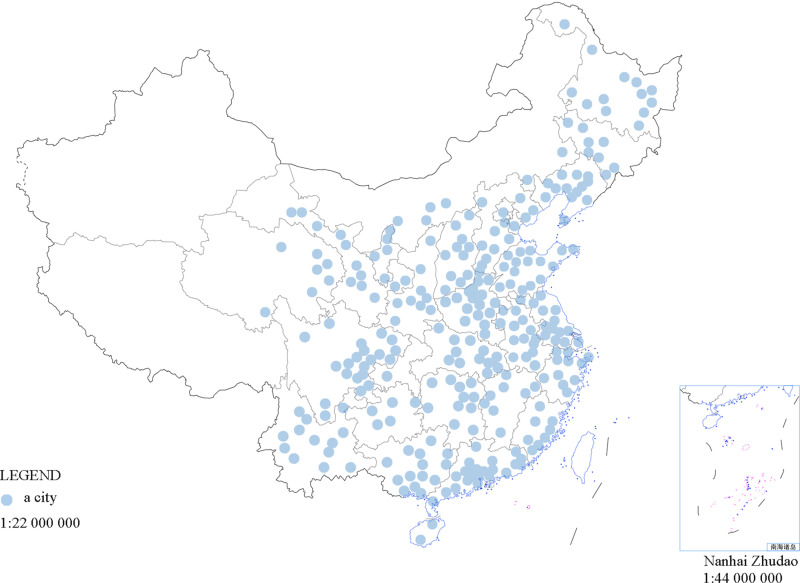
Distribution of 284 sample cities in China. Each point represents a city.

The obtained Weibo data were subsequently cleaned. On the one hand, for Weibo text data, we remove large-scale references to # and URL external links, multiple forwards of the same Weibo text, and Weibo texts with obvious features posted by ‘internet army’ or with machine account characteristics [41,42]. These Weibo texts are usually not intended to express true human feelings or opinions, but rather to influence public opinion or promote advertisements [43]. On the other hand, most emotional Weibo texts are published without geographic tags, which is a feature provided by Weibo that allows users to freely choose whether to display their current real location when posting. Considering that residents with a tendency toward depression are less willing to disclose personal information when posting on Weibo [5], geographic tags may be ignored when publishing depression messages; nevertheless, the emotional expression of this group of people cannot be ignored. Therefore, this study first used samples with geographic tags as Weibo data for the city, and then used Weibo samples with user registered address information as Weibo data for the city, removing samples for which neither user-registered address information nor Weibo geographic tag information was available. After cleaning, 5.59 million Weibo user registration information and 8.54 million valid Weibo texts were obtained from 284 prefecture-level cities in 29 provinces (autonomous regions, and municipalities) from January 1, 2016, to December 31, 2019. The distribution of the sample cities is shown in Fig 1. The specific names of 284 prefecture-level cities and their respective provinces (autonomous regions, and municipalities directly under the central government) can be found in S5 Appendix.

### 2.2. Measures and descriptions of the key variables

#### 2.2.1. Explained variable.

We utilized the depression index as the explained variable. This variable quantifies the likelihood and potential risk of depression among residents of a city. The depression values of each Weibo text obtained by all users from relevant posts in each city daily were calculated at the city level to obtain the total daily depression tendency value for each city. To avoid the influence of a skewed distribution of the data, we used the logarithm of the total depressive tendency value as the explanatory variable. The calculation of the depression value of Weibo posts constituted the technical key of this study, for which we referred to existing research methods [44–46]. By constructing a dictionary of depression emotions, natural language processing technology was used to calculate the depression value of the Weibo text. The main calculation process and methods are described in detail in S1 Appendix.

(1) Construction of the depression dictionary

We constructed a dictionary of depressive emotions on the basis of depression scales, Weibo super-topics, and existing Chinese dictionaries. The dictionary included emotional words, degree adverbs, and negative words [44–46].

The emotional words for depression were initially extracted from the six commonly used depression scales in the international diagnosis of depression. After duplicate words related to depression symptoms from these scales were eliminated, 132 depression seed words, such as “depression”, “silence” and “fatigue”, covering psychological characteristics, physical symptoms, and behavioral performance were obtained. Since network users tend to use synonyms or network terms to express their emotions, unlike formal medical diagnoses, the use of only seed words for text quantitative evaluation can cause measurement distortion [47]. Therefore, we expanded the depression dictionary through Weibo super-topic extraction and Chinese synonym tools.

To facilitate users with shared interests to convene for interactive discussions on a unified theme, Weibo introduced the ‘super-topics’ online community function [48]. When a blog entry is posted on a super-topic, the post contains the ‘#depression#’ logo and is displayed on the topic page. We obtained the Weibo post text under the ‘depression’ super-topic and used natural language processing methods, such as text rank and term frequency-inverse document frequency (TF-IDF), to extract high-frequency keywords as Weibo depression feature words. We then used the Python Chinese synonym toolkit ‘synonyms’ to obtain synonyms for the abovementioned depression seed words and the Weibo depression feature words. After manual verification, we obtained a total of 653 basic depression-related words. Following the methods of D et al. [49] we constructed the depression dictionary for this study, which included the basic words related to the depression emotion, negative words obtained from the ‘data hall’, and degree adverbs obtained from the ‘How Net’ lexicon. Each word in the Depressive Emotion Dictionary was marked as the next Weibo text depression value calculation, which was based on the Depression Diagnostic Scale and the standard configuration weights and scores of negative words and degree adverbs (see S2 Appendix for the dictionary).

(2) Depression value calculation

We used a word segmentation tool to categorize the words in each Weibo post. Then we calculated the depression value according to the weight and score of various words in the dictionary. The calculation rule is as follows:


dep=∑i=1n−1^Nn×Ag×Dw×Es (1)

where *dep* is the depression value of each Weibo post, *Nn* is the number of negative words, *Ag* is the degree level of the degree of the adverbs, *Dw* is the weight of each word in the depression dictionary, *Es* is the score of the emotional words, *i* is an emotional word, and *n* is the number of all emotional words in the post.

*Ag* is divided into six types of degree adverbs ‘extreme or most, super, very, relatively, slightly, or owe’, assigned values of 2, 1.75, 1.5, 1, 0.5, or 0.25, respectively. The *Dw* calculation assigns weights ranging from 1 to 7 to different categories of words in the constructed depressive lexicon according to the core characteristics of major depressive disorder. *Es* directly obtains the assignment score of the word in the BosonNLP sentiment dictionary. The BosonNLP sentiment dictionary is a mature and open-source Chinese sentiment dictionary. It assigns a score for each word in the dictionary (ranging from -7 to 7). The higher the score, the more positive the emotion. The dictionary contains numerous internet jargon and covers a wide range of Weibo words. It has been frequently used for sentiment analysis and serves as a fundamental sentiment score dictionary for calculating depression [48].

For the convenience of observation, when the calculation of each Weibo post was completed, the *dep* results were multiplied by (-1), using a sigmoid function to achieve 0-1 normalization, and then multiplied by 10 to produce a value range of 0-10; the greater the value is, the higher the level of depressive tendencies is [49]. The total depression tendency value must be added to the depression values of all Weibo posts in that city on that day.

(3) Verification of the calculation method for the depression value

To verify the reliability and stability of our calculation results, this study selected the Baidu Brain AI open platform to calculate the probability of emotional polarity for 5% of the study samples. Baidu Brain AI (https://ai.baidu.com/tech/nlp_apply/sentiment_classify) is an AI open platform developed by Baidu, a famous Chinese internet company, which can automatically determine the emotional polarity category of text and assign a positive or negative probability value of emotional polarity to each paragraph; however, it cannot refine the judgment of the text’s tendency toward depression. We followed the method of Zhiwei et al. [50] and selected the results of 5% samples of depression values to verify them with the results generated by the negative emotion calculation with the Baidu Brain AI open platform. The analysis revealed a significant correlation between the two calculated results (r = 0.25, p < 0.01), indicating the stability and reliability of our depression value calculation method.

#### 2.2.2. Explanatory variable.

To measure air pollution, this study used PM2.5 as the key explanatory variable and the daily air quality index (AQI) as an alternative measure of air pollution to test the robustness of the results. The daily average PM2.5 concentration (μg/m^3^) and AQI in each city were obtained from the website of the China Environmental Monitoring Station (http://www.cnemc.cn/).

#### 2.2.3. Control variables.

In addition to air quality, other factors, such as local economic and social conditions, as well as natural conditions, such as weather, can influence the onset of depression [51]. Therefore, the evaluation included several control variables for each city from 2016 to 2019, including year-end population, GDP, annual per capita disposable income of urban residents, maximum temperature, minimum temperature, wind speed, wind direction, weather conditions, holidays, and seasonal effects. The three annual variables of the year-end population, GDP and annual per capita disposable income of urban residents in each city are directly used as daily data. The data are compiled from the statistical yearbooks of each city. Weather data such as temperature and wind direction data are daily data that can be matched with other daily data. The weather data are obtained from daily weather forecasts from the website of the China Meteorological Administration (https://weather.cma.cn/).

#### 2.2.4. Descriptive data analysis.

The definitions and descriptive statistics of all the variables of the sample are shown in Table 1. As shown in Table 1, the average daily PM2.5 concentration in the sample cities during the study period was 41.44 μg/m^3^, which is 18.4% higher than 35 μg/m^3^, the annual average concentration of PM2.5 specified in the Chinese ‘Environmental Air Quality Standards’. This standard value is much greater than that of the World Health Organization’s ‘Air Quality Guidelines’ published in 2014. The guidelines recommend an annual average PM2.5 concentration of 10 μg/m^3^ or less to ensure good air quality [52].

**Table 1:** Variable indicator description.

Type	Variable	Description	Observations	Mean	Sd	Min	Max
Dependent Variable	lndep	The logarithm of the 24-hour overall depression tendency value of prefecture-level cities	349366	3.80	1.68	-23.03	9.21
Explanatory Variable	pm25	The average concentration of PM2.5 pollutants in 24 h of prefecture-level cities (μg/m^3^)	349366	41.44	34.06	0	698
	AQI	Air quality index (AQI)	349366	70.98	41.93	0	500
Control Variables	Pop	Year-end resident population (ten thousand)	349366	425.36	362.78	13.9	3330
	GDP	Gross domestic product at the end of the year (billion yuan)	349366	328.13	444.68	3.19	3815.53
	Income	Annual per capita disposable income of urban residents (ten thousand yuan)	349366	3.47	0.89	1.61	7.38
	Tmax	Daily maximum temperature (°C)	349366	20.38	10.81	-26	42
	Tmin	Daily minimum temperature (°C)	349366	10.94	11.45	-41	32
	Wprat	Wind speed (m/s)	349366	3.60	2.20	0.9	32.68
	Wind	Wind direction (no wind = 0, east = 1... southwest = 8)	349366	3.10	2.72	0	8
	Weather	Weather conditions (clear = 1, cloudy = 2... rain = 6)	349366	2.42	1.19	1	6
	Holiday	Working days = 0, holidays = 1	349366	0.29	0.45	0	1
	Season	Spring = 1, Summer = 2, Autumn = 3, Winter = 4	349366	2.50	1.12	1	4
Instrumental Variable	iv_negh	Weighted pollutant values of surrounding cities: weighted sum of PM2.5 concentrations of surrounding cities for the day	335057	0.58	0.73	0	15.08

Note: Due to the lack of pollution values for the surrounding cities of individual samples, individual samples were missing after instrumental variable calculation, and instrumental regression is based on instrumental variable samples.

**Fig 2:**
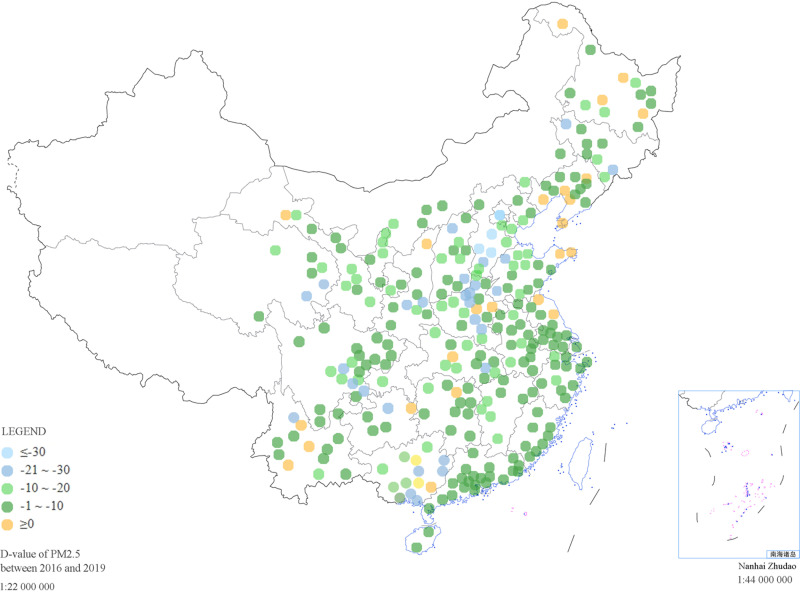
Annual average PM2.5 differences in sample cities between 2016 and 2019. According to the legend, each point represents a city, and the color ranges from sky-blue to orange. A color closer to orange indicates that the PM2.5 concentration in the city in 2019 was greater than that in 2016. A negative value indicates that the PM2.5 concentration decreased over the four years.

Since 2016, the Chinese government has implemented *the Air Pollution Prevention and Control Law*. In June 2018, the State Council issued the *Three Year Action Plan for Winning the Blue Sky Defense War*, which began systematic air pollution control [53]. As a result, the air pollution situation has been fundamentally reversed. Fig 2 shows a distribution map of the differences between the average annual concentrations of PM2.5 pollutants in the sample cities in 2019 and those in 2016. A color closer to orange indicates that the PM2.5 concentration in the city in 2019 was greater than that in 2016. A negative value indicates that the PM2.5 concentration decreased over the four years. Fig 2 shows that 91.5% of the 284 cities had a negative difference. Some cities demonstrated positive differences in pollutant concentrations because the air quality of these cities has been excellent. For example, the PM2.5 concentration in Yichun in 2019 was 3 μg/m^3^ higher than that in 2016, marked in orange in Fig 2, and it was still only 19.4 μg/m^3^ in 2019. These results indicate that although the annual average daily concentration of PM2.5 in China was higher than the Chinese national standard of 35 μg/m^3^, air pollution control measures have been very successful. Similar to existing research findings, China’s efforts to combat air pollution have improved in air quality [54].

**Fig 3:**
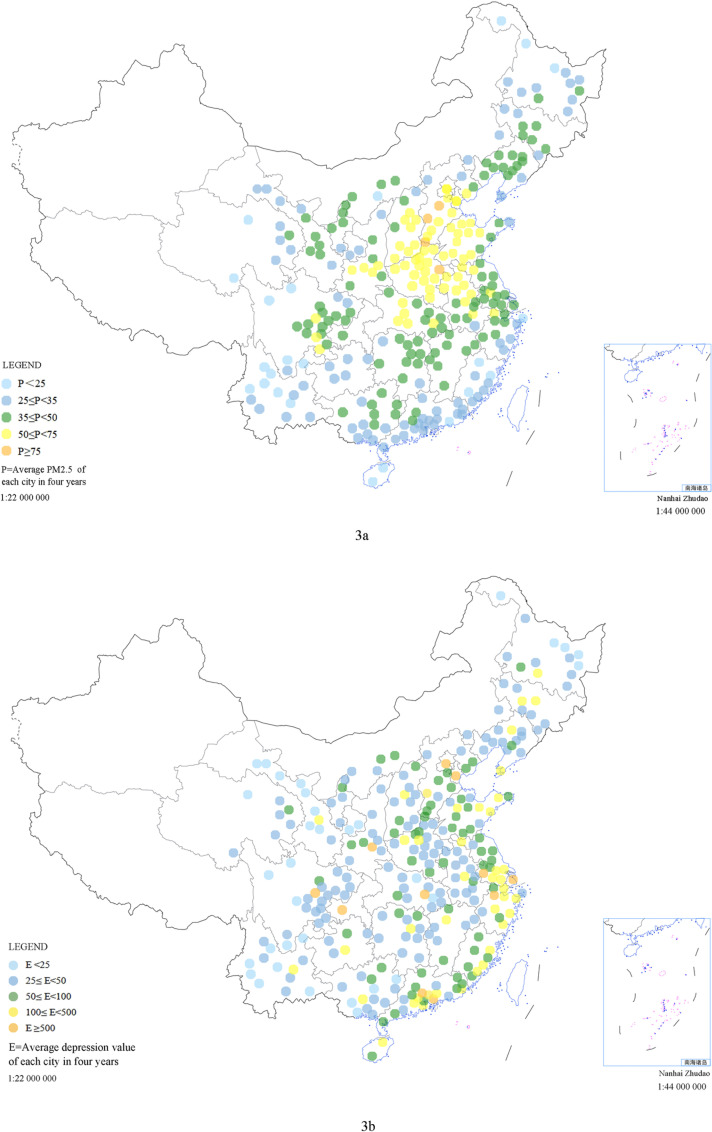
Annual average distributions of PM2.5 (3a) and total depressive tendency values (3b) in the sample cities from 2016 to 2019. Each point represents a city. The annual average concentration distribution of PM2.5 in sample cities from 2016 to 2019, with the left legend indicating the range of the annual average concentration of PM2.5; the larger the value is, the greater the concentration of PM2.5, and one color represents the annual concentration range of PM2.5. If the average annual concentration of PM2.5 in a certain city is less than or equal to 25 μg/m^3^, the city is marked in sky-blue in Fig 3a, and if the annual average concentration of PM2.5 in the city is greater than or equal to 75 μg/m^3^, it is marked in orange. Fig 3b shows the average 4-year distribution of the total depressive tendency in the sample cities from 2016 to 2019. The numbers represent the total depressive tendency values, and different colors represent different ranges of the total depression tendency values. The larger the value is, the greater the depressive tendency.

Fig 3 presents a visual representation of the average PM2.5 concentration and total depressive tendency in 284 cities across 29 provinces from 2016 to 2019. Part 3a indicates higher PM2.5 concentrations, which have been notably higher in Beijing-Tianjin-Hebei, Henan, and Shandong in recent years. Part 3b represents the average total depressive tendency value over the four years. There was a significant difference in total depressive tendency before the logarithm was taken. The larger the value, the more significant the depressive mood tendency. The graph shows that the depressive mood tendencies of people in first-tier cities such as Beijing, Tianjin, Shanghai, and Guangzhou, as well as in developed coastal areas, are relatively significant. However, the distributions of in other areas with less significant depressive tendencies in Yunnan, Qinghai, and Northeast China are similar to the air pollution distribution.

### 2.3. Estimation strategy

To evaluate the impact of air pollution on depression, this study employed an OLS regression model for empirical estimation. The benchmark equation is defined as follows:


$$lndep_{it}=\beta_{0}+\beta_{1}P_{it}+\beta_{2}X_{it}+\varepsilon_{it}$$ (2)

where *lndep*_*it*_ is the logarithm of the depression value of city *i* on day t. The expansion of the measurement unit does not affect the model fit [55]. To increase the convenience level for presenting and interpreting the results, the dependent variable value was expanded by 100 times. *P*_*it*_ is the pollution level of city *i* on day *t*; and *X*_*it*_ is a series of control variables for city *i*, including population, GDP, per capita income, daily temperature, wind speed, wind direction, weather conditions, holidays, and seasonal effects. $\varepsilon_{it}$ is the random error term.

Due to the endogeneity issue between air pollution and depression, whereby air pollution can harm individuals’ physical and mental health, leading to depression, and individuals with depression may reduce their activity and productivity levels, causing a reduction in work-related pollutants, existing research has employed the instrumental variable method. One commonly used approach is the air pollution spillover effect from upwind areas, which measures the pollutant weighting of surrounding cities [20,56]. The instrumental variable should be strongly correlated with local pollution but weakly correlated with other unobservable variables [57]. To address this issue, this study employs the weighting of pollutants in surrounding cities as an instrumental variable, which calculates the weighted sum of PM2.5 concentrations in cities surrounding the sample on the same day. The instrumental variable calculation and regression model are presented below:


$$P_{it}=\delta_{0}+\delta_{1}I_{it}+\delta_{2}X_{it}+\gamma_{i}+\mu_{i}$$ (3)

$$lndep_{it}=\beta_{0}+\beta_{1}P_{it}+\beta_{2}X_{it}+\gamma_{i}+\varepsilon_{i}$$ (4)

where Equation (3) is the first-stage model of the instrumental variable; *P*_it_ is the air pollution value obtained by the instrumental variable; *I*_it_ is the pollution-weighted value of the cities surrounding city *i* on day t; and γ_i_ is the fixed effect of city *i*. The remaining variables have the same meanings as those described for Equation (2). Equation (4) is the second-stage model of the instrumental variables.

According to the literature [57,58], the instrumental variable can be calculated as follows (S3 Appendix shows the method of calculating the instrumental variable for details):


$$I_{it}=\sum_{j}\left(W_{ijt}\times pe_{jt}/d_{ij}\right),100\;km<d_{ij}<200\;km$$ (5)

where *I*_it_ is the pollution-weighted value of the surrounding cities of city *i* on day t; *pe*_jt_ is the PM2.5 concentration of the surrounding city *j* on day t; and *d*_ij_ is the distance between city *i* and city *j*. We set the distance range between 100 km and 200 km. The weight *W*_ijt_ is based on the cosine of the difference between the azimuth angles of city *j* and city *i* and the wind direction angle of city *i* on day *t* (the north wind direction is set to 0 degrees, the northeast wind direction is set to 45 degrees, and so on). If the surrounding city *j* is upwind of city i, then the cosine of the angle difference is the weight; when the cosine is negative, then city *j* is downwind of city *i*, and the weight is set to 0. The distance and azimuth in Equation (5) were calculated according to the latitude and longitude of the sample cities, which were derived from the Baidu map picking coordinate system.

## 3. Results and discussion

### 3.1. Influence of air pollution on depression

The results of the OLS and IV regressions are presented in Table 2. Column (1) shows the estimation without any control variables based on OLS equation (2), whereas Column (2) shows the results after introducing all other control variables. Both coefficients of PM2.5 shown in Columns (1) and (2) are positive and significant at the 1% level, indicating that air pollution significantly affects people’s depression. The coefficients suggest that with every 1 μg/m^3^ increase in the PM2.5 concentration of a city, the depression of local residents’ increases by 0.0437%-0.0495%.

**Table 2:** Impact of air pollution on residents’ depression.

Variables	OLS		IV			
	(1)	(2)	(3)	(4)	(5)	(6)
pm25	0.000437^***^ (5.63)	0.000495^***^ (6.57)	0.000395^***^ (3.23)	0.00197^***^ (19.94)	0.000402^***^ (3.35)	0.000559^***^ (4.59)
Pop		0.00568^***^ (270.59)		0.00570^***^ (237.30)	0.00569^***^ (236.66)	0.00569^***^ (236.65)
GDP		0.00178^***^ (42.53)		0.00178^***^ (42.81)	0.00177^***^ (42.56)	0.00177^***^ (42.49)
Income		-0.476^***^ (-45.83)		-0.478^***^ (-42.00)	-0.468^***^ (-40.55)	-0.467^***^ (-40.44)
Tmax		-0.00100 (-1.18)			0.00138 (1.56)	-0.00104 (-1.13)
Tmin		-0.0117^***^ (-13.31)			-0.0123^***^ (-12.87)	-0.0115^***^ (-11.91)
Wprat		0.0152^***^ (12.11)			0.0168^***^ (14.25)	0.0154^***^ (13.05)
Wind		-0.00958^***^ (-8.74)			-0.00904^***^ (-8.59)	-0.00885^***^ (-8.41)
Weather		0.000872 (0.39)			0.00523^**^ (2.30)	0.00152 (0.66)
Holiday		-0.00748 (-1.59)				-0.00767 (-1.62)
Season		-0.0282^***^ (-12.21)				-0.0289^***^ (-12.57)
constant	2.656^***^ (57.57)	3.303^***^ (66.52)	2.658^***^ (38.77)	3.230^***^ (44.80)	3.172^***^ (43.29)	3.275^***^ (44.30)
Fixed effect	YES	YES	YES	YES	YES	YES
N r2 F	349366 0.273 460.5	349366 0.444 949.7	335057 0.278	335057 0.452	335057 0.456	335057 0.456

Note:

* ,

** ,

***  represent significance at the 0.1, 0.05, and 0.01 levels, respectively. The corresponding standard errors are in parentheses.

Columns (3) to (6) display the results estimated by the IV model. The results in Column (3) are estimated without any control variables, whereas Columns (4)-(6) display the estimated results of sequentially adding control variables such as economic and social conditions, weather, season, and holiday effects. All the coefficients of the key variables are significant at the 1% level throughout Columns (3) - (6). Compared with the OLS regression results, the coefficient of PM2.5 increased slightly based on the IV regression results (0.0495% and 0.0559%, respectively), indicating that the OLS approach underestimates the impact of PM2.5 on residents’ depression. These findings are consistent with those of previous studies on the relationship between air pollution and well-being. Zheng et al. reported that the PM2.5 concentration (or the AQI) increased by a standard deviation, whereas the happiness index decreased by 0.043 (or 0.046) standard deviation based on 2014 Weibo data, demonstrating that an increase in pollutant concentration inhibits people’s happiness [20]. We further confirmed that for every 1 μg/m^3^ increase in the PM2.5 concentration, the depression of residents increased by 0.0559%. The close relationship between air pollution and people’s depressive symptoms further explains how air pollution reduces happiness. It should be noted that Weibo user group as the representative of the majority of netizens, has a high proportion of youthfulness and posts are mainly published in cities. This characteristic may result in the emotional expression of the elderly, rural users, and those with low frequency of social media use being overlooked. Previous studies have shown that the elderly population is more sensitive to air pollution [59]. Therefore, we believe that there is a possibility of underestimating the impact of air pollution on depressive emotions in this study.

### 3.2. Cumulative effects of air pollution on depression

Medical evidence has shown that air pollutants can lead to depressive symptoms by causing oxidative damage and inflammation in the nervous systems. This inflammatory response may gradually increase over time and may also be weakened by activating self-protection mechanisms. To determine which scenario is more likely to occur, we assessed the long-term cumulative effect. We converted the daily depression values into weekly, monthly, quarterly, and annual averages for each city. Other explanatory, instrumental, and control variables were also converted into the corresponding averages, and instrumental variable regression was conducted.

The results are presented in Table 3. The results indicate there are weekly cumulative effects of the urban PM2.5 concentration on residents’ depression, which are significant at the 0.01 level. For every 1 μg/m^3^ increase, the weekly tendency toward depression in urban residents increased by 0.077%. Moreover, the concentration of PM2.5 in cities was found to have monthly, quarterly or annual cumulative effects on residents’ depression, but these effects were not significant. This finding suggests that even if the number of days with high pollution levels decreases, the accumulation of fine pollutants over a period still has a close positive correlation with depression. A high concentration of pollutants over a short period may not necessarily result in immediate morbidity or death; however, after a certain threshold is reached, harm can be caused to the human body, especially in the case of short-term continuous pollution.

**Table 3:** Cumulative effects of air pollution on depression.

Variable	Weekly	Monthly	Quarterly	Annual
pm25	0.000770^***^ (3.61)	0.000606 (0.94)	0.00105 (0.72)	0.0129 (0.57)
Control variable	YES	YES	YES	YES
Fixed effect	YES	YES	YES	YES
N	56151	12963	4336	1084
R^2^	0.741	0.801	0.814	0.837

### 3.3. Robustness test

Air pollution may have a threshold effect on neurological damage, meaning that it may cause harm only after a certain level of absolute pollution is reached. In this study, we analyzed data from 284 cities with varying levels of absolute pollution and different economic and social conditions. To eliminate the possible influence of extreme values, we removed any samples with outliers in air quality and verified the robustness of our results. Additionally, in the IV estimation, we used pollutants within a radius of 100 to 200 km as instrumental variables, which may have ignored the impact of pollution sources that are closer or farther away. Therefore, we also tested the robustness of our method by adjusting the radius distance.

According to the considerations mentioned above, we conducted robustness tests by replacing independent variables with the AQI, selecting urban subsamples with different conditions, randomly selecting samples, and replacing instrumental variables with different radius distances. The test results are presented in Table 4. Model (1) shows the results of the instrumental variable regression with the AQI as an independent variable. The AQI is an air quality index, and higher values indicate more severe air pollution. The regression coefficient remains the same even after the independent variables are changed, indicating that as the AQI increases, people’s emotions tend to become more depressed. Model (2) adjusts the distance *d*_*ij*_ of the weighted value of pollutants in the surrounding cities of the instrumental variable to 150 km < *d*_*ij*_ < 250 km. After recalculating the instrumental variable, the coefficient of the pollution variable remains significant.

**Table 4:** Robustness test results.

Variable	(1)	(2)	(3)	(4)
	AQI	Alternative IV	Subgroup cities with moderate air quality	80% subsample randomly selected from sample cities
pm25	–	0.000872^***^ (6.69)	0.000288^*^ (1.86)	0.000642^***^ (4.94)
AQI	0.000480^***^ (4.59)	–	–	–
Pop	0.00569^***^ (236.64)	0.00566^***^ (236.46)	0.00589^***^ (202.37)	0.00580^***^ (214.28)
GDP	0.000177^***^ (42.47)	0.000187^***^ (45.01)	0.000169^***^ (35.07)	-0.0000690^***^ (-5.04)
Income	-0.466^***^ (-40.27)	-0.487^***^ (-41.50)	-0.567^***^ (-42.40)	-0.0000271^***^ (-23.28)
Tmax	-0.00147 (-1.56)	-0.00179^ * ^ (-1.94)	-0.000874 (-0.82)	-0.00190^ * ^ (-1.83)
Tmin	-0.0111^***^ (-11.30)	-0.0105^***^ (-10.92)	-0.0125^***^ (-11.35)	-0.0106^***^ (-9.91)
Wprat	0.0149^***^ (12.82)	0.0162^***^ (13.67)	0.0161^***^ (11.55)	0.0169^***^ (12.92)
Wind	-0.00889^***^ (-8.44)	-0.00918^***^ (-8.50)	-0.00968^***^ (-8.16)	-0.00932^***^ (-8.03)
Weather	0.00205 (0.89)	-0.000501 (-0.22)	0.00201 (0.76)	-0.000581 (-0.23)
Holiday	-0.00770 (-1.63)	-0.00833^ * ^ (-1.75)	-0.00536 (-0.99)	-0.00302 (-0.58)
Season	-0.0281^***^ (-12.35)	-0.0299^***^ (-12.85)	-0.0288^***^ (-10.92)	-0.0304^***^ (-12.00)
Constant	3.265^***^ (44.07)	3.324^***^ (44.82)	3.510^***^ (46.10)	2.908^***^ (35.29)
Fixed effect	YES	YES	YES	YES
N R^2^	335057 0.456	341947 0.445	269266 0.432	268052 0.458

Using the composite index of ambient air quality, ‘The China Eco-Environmental Status Bulletin’ (hereafter referred to as ‘The Environmental Bulletin’) lists cities with relatively good or poor air quality [60]. Using data from ‘The Environmental Bulletin’ from 2016 to 2019, for each year, we selected 10 cities with relatively good air quality and 10 cities with relatively poor air quality. We removed 56 cities after the deduplication process, and ultimately selected 228 cities with intermediate air quality as subsamples for the robustness test. The regression results are found to be significant in Model (3) based on the IV method. In Model (4), we randomly select 80% of the samples for IV regression, and the key coefficient was found to be significant, which is consistent with the results of Table 2. These four results demonstrate that the findings of this study are robust, indicating that as air pollution worsens, and residents tend to experience higher levels of depression.

### 3.4. Heterogeneity analysis

The stress response to pollution can vary across different economic and social environments. Previous studies have demonstrated that the health impacts of air pollution vary by pollution levels and factors such as the availability of heating systems in cities during the winter months, which can have different consequences for physical and mental health [59]. Additionally, individuals with different income levels may have distinct perceptions of pollution [61]. Examining the heterogeneous effects of air pollution on depression can provide a scientific basis for developing targeted interventions. Therefore, we evaluated the heterogeneity of the impact of air pollution on residents’ depression across different regional cities, pollution periods, and economic conditions. The results of this analysis are presented in Table 5.

**Table 5:** Heterogeneity analysis of different subsamples.

Variable	(1) Heating	(2) No heating	(3) Weekday	(4) Holiday	(5) Lower income	(6) Higher income
pm25	0.00101^***^ (5.46)	0.000315^ * ^ (1.91)	0.000800^***^ (5.49)	0.0000113 (0.05)	0.00100^***^ (5.83)	-0.0000939 (-0.59)
Control variable	YES	YES	YES	YES	YES	YES
Fixed effect	YES	YES	YES	YES	YES	YES
N R^2^	116839 0.416	218218 0.478	239494 0.458	95563 0.454	160718 0.278	174339 0.562

To assess the impact of air pollution on residents’ depression levels, accounting for the presence or absence of systematic urban heating facilities, which is a key factor affecting the degree of air pollution during the heating season is crucial. This season typically extends from mid-November to mid-March of the following year. Thus, the net effects of these two factors need to be empirically tested. Therefore, we divided our sample into two subgroups, namely, cities with heating facilities and cities without heating, and conducted a regression analysis. As shown in Columns (1) and (2) of Table 5, air pollution was found to have a significant effect on residents’ depression in both subgroups. Similar to the results of previous studies [59], urban residents with heating have a slightly stronger response to changes in air pollution than residents without heating.

Residents have different commuting routes on weekdays, weekends, or holidays, which may lead to different levels of exposure to pollution. After controlling for weather and other related variables, the results in Columns (3) and (4) suggest that residents’ depressive tendencies on weekdays are significantly influenced by air pollution, whereas no significant impact is observed on holidays. Perhaps during smoggy weather on holidays, people may be more likely to take measures to avoid outdoor exposure, thus reducing the mental harm caused by air pollution.

Residents with varying income levels exhibit varying sensitivities to air pollution. To assess heterogeneity in this situation, we divided the samples into two subgroups according to the median annual income of residents, namely, subgroups in lower or higher-income cities. The evaluated results indicate that air pollution significantly impacts the depression levels of residents in lower-income cities, whereas the depression of residents in high-income cities is not significantly affected by air pollution. This disparity may be attributed to the ability of high-income groups to select transportation options unaffected by adverse weather conditions or to acquire air purifiers and other tools that mitigate the effects of air pollution [62,63].

### 3.5. Placebo test

**Fig 4:**
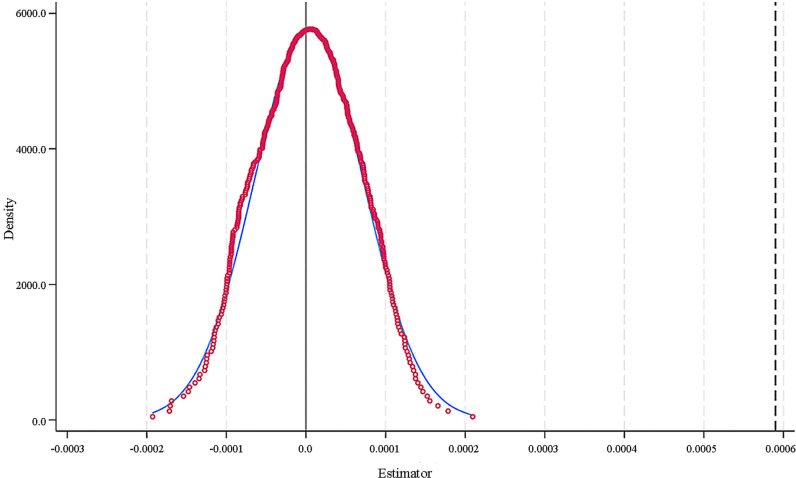
Distribution of the estimated coefficients of the PM2.5 regression after random assignment.

To eliminate the potential influence of other omitted variables on the results, we conducted a placebo test by randomly assigning the independent variable PM2.5 to sample cities and running the instrumental variable regression 500 times to obtain the coefficient estimates [64]. As shown in Fig 4, the placebo regression coefficients were concentrated near 0 and far from the actual regression coefficient of 0.000559, indicating that our estimated result is robust.

## 4. Monetization of mental health benefits

If the depression caused by air pollution is not treated in a timely manner, it can affect people’s physical functioning and even family and social functioning, thereby increasing the economic burden on individuals and families and generating medical costs [65].

According to the results of this study, for every 1 μg/m^3^ increase in PM2.5 due to air pollution, the likelihood of depression among residents increases by 0.0559%. To determine the medical cost related to depression caused by air pollution, we need to determine the medical cost of each patient with depression. Moreover, inpatient costs account for the largest proportion of direct medical expenses associated with depression [66]. Therefore, this study focused on analyzing the inpatient costs to represent medical costs resulting from depression and to evaluate mental health benefits. Due to the age distribution of the Weibo population [32] and because hospitalization costs for elderly patients with depression are significantly greater due to their complex physical health [67], medical expenses for elderly people are not considered in this study.

Table 6 presents the results of various studies on the inpatient costs of depression. Luppa et al. conducted a review of 24 relevant studies and reported that the annual inpatient costs of depression range from $1,000 to $2,500 in developed Western countries [66]. In Australia, the per capita direct cost of hospitalization for depression has been estimated at $2454 [68]. In the United States, the cost of hospitalization for employees has been estimated at $1341 [69]. Yawei et al. analyzed medical data from 488 children with depression in Shandong, China, and reported that the average inpatient cost was $1829 [70]. Compared with Western countries, China’s medical expenses for depression patients lack medical service costs and the country has more public than private hospitals, resulting in lower costs. The majority of Chinese patients with depression receive medical treatment at public hospitals. According to data from the China Health Statistics Yearbook, the per capita medical expense for discharge from a public hospital for someone with a mental or behavioral disorder in 2019 was 7952.52 yuan (or $1172.2, calculated using a currency exchange rate of 6.87 RMB to 1 USD) [71]. Thus, according to the proportion of cases of depression, the per capita medical cost of depression in China’s public hospitals is estimated to be 1988.13 yuan ($295), which is the minimum medical expenditure for depression.

**Table 6:** Costs of medical expenditures for depression.

Study/Source	Region	Research object	Costs (US$)
Luppa et al. 2007	Western developed countries	Adults (18-65)	1000-2500
Druss et al. 2000	America	Adults (18+)	1341
Hawthorne et al. 2003	Australia	Adults (15+)	2454
Ma et al. 2021	Dalian, China	Adults (40-68)	651
Yawei et al. 2019	Shandong, China	Children (18-)	1829
China Health Statistics Yearbook. 2019	China	all	295

Therefore, per the abovementioned statistics and research data, the per capita direct cost of depression in China ranges from approximately $300 to $2500, which is equivalent to 2061 yuan to 17175 yuan. According to the existing research [7] and the formula (the detailed process can be found in S4 Appendix), the per capita medical costs of depression caused by air pollution ranges from $0.17 to $1.4 (from 1.17 to 9.62 yuan). Since the sample of this study is Weibo users, not all age groups of Chinese will use Weibo. Therefore, in combination with the age distribution of Weibo users and depression patients investigated in previous studies [23,72,73]. The sample of this study can estimate that the proportion of depression patients is about 45%-86%. While 95 million people in China suffer from depression, only 8.2% of depressed patients in China actively seek medical treatment [74]. If 8.2% of depression patients receive treatment, then the estimated cost of mental health care ranges from $0.59 million to $9.36 million annually. From 2016 to 2019, the average annual concentration of PM2.5 in China decreased by 8.45 μg/m^3^. During this period, due to the decrease in air pollution, the reduction in per capita annual hospitalization expenses ranged from $1.42 to $11.81. The average annual cost for reducing medical expenses for depression nationwide due to improved air quality ranges from $4.97 million to $79.12 million in China, indicating that the improved air quality results in significant economic benefit. These results are lower than that of previous research [7]. We supposed it was the bottom bound of the benefit because the research sample are netizens, which would lead a possible underestimating medical expenditures.

## 5. Conclusions

Depression is a prevalent and burdensome disease that imposes a heavy economic burden on society and families. While previous studies have shown a significant association between short-term air pollution exposure and the incidence of depression, the cumulative effects of mid- and long-term exposure remain controversial. This study employs big data and text econometric analysis techniques to construct a social media depression dataset consisting of 8.54 million observations across 284 prefecture-level cities in China from 2016 to 2019. Therefore, by utilizing the upwind air pollution spillover effect of air pollution as an instrumental variable, we investigate both the short-term and long-term effects of air pollution on residents’ depression. We also analyze the heterogeneity and adjustment factors of the impact, considering factors such as per capita income levels and holiday pollution avoidance.

First, both short- and mid-term air pollution are closely associated with the exacerbation of residents’ depression. Specifically, every 1 μg/m^3^ increase in the daily average PM2.5 concentration in the city results in a 0.0559% increase in residents’ tendency toward depression. While a 1 μg/m^3^ increase in the weekly average PM2.5 concentration leads to a 0.077% increase. Second, the robustness analysis revealed that the impact of air pollution on depressive symptoms remains significant even when the urban sample selection in each dimension is changed. Furthermore, while some avoidance measures could reduce the harmful effects of air pollution on people’s mental health, they could also exacerbate health inequality among people with different socioeconomic conditions. From 2016 to 2019, the average PM2.5 concentration in Chinese cities decreased by 8.45 μg/m^3^, resulting in a cumulative reduction in medical costs ranging from $4.97 million to $79.12 million. This finding provides further evidence of the significant economic and social benefits of Chinese environmental governance policies.

This study’s findings indicate that air pollution has significant and persistent negative effects on mental health. Even if the number of days with severe air pollution decreases in the short term, the cumulative impact of pollution on people’s depression still exists. Given that the research sample is netizens, there is a possibility of underestimating the results of this study. The Chinese government’s ‘Fight for Blue Skies’ policy has achieved remarkable results in reducing the burden of depression-related medical costs. Thus, it is essential to continue our efforts to reach healthy and liveable air quality standards as rapidly as possible. While strengthening environmental pollution control, it is also necessary to develop protective policies for people with different economic and social conditions to reduce inequality in environmental pollution injuries, such as reducing outdoor work hours in specific industries during polluted weather, or providing necessary protective conditions and air pollution subsidies. Furthermore, the government should focus on addressing depression. First, there is a need to enhance public education and awareness of scientific approaches to managing depression. This empowers individuals to regulate their emotions when experiencing depressive tendencies. Second, government agencies can harness big data capabilities to promptly identify individuals exhibiting signs of depression through online surveillance, and implement proactive interventions to prevent the worsening of their condition.

This study has some limitations. First, although this study considered the low self-exposure of patients with depression and combined Weibo geographic tags and registration addresses to reduce the error of Weibo users’ addresses, not fully using geographic tag addresses would reduce the accuracy of Weibo city positioning. Future research using data with geographic position information will allow us to improve it. Second, we realize that the Weibo user group deviates from representing the entirety of China’s population and may not fully capture the actual circumstances of all residents. Nonetheless, under current conditions, Weibo users provide the largest accessible sample for addressing the subjective biases inherent in self-reported data used in previous studies. Looking ahead, the development of more extensive and inclusive national data sets in China would enable further comprehensive and in-depth research, advancing and refining the robustness of our findings. In addition, owing to data availability, this study did not obtain Weibo users’ personal information, such as age, occupation, and avoidance behavior. In the future, we can further explore the relationship between the cost of self-protection and mental health. And we also hope to access to data on mental health clinics and treatment costs in prefecture city or county level so that we can also accurately evaluate the cost-benefit of air pollution prevention and control in mental health.

## Supporting information

S1 Appendix Depression calculation method. (PDF)

S2 Appendix Depression basic words. (PDF)

S3 Appendix Method for calculating instrumental variables. (PDF)

S4 Appendix Monetization of mental health benefits. (PDF)

S5 Appendix 284 prefecture-level cities and their respective provinces. (PDF)
