# Supplementary material for: The impact of fine particulate matter on depression: Evidence from social media in China
Source: PLoS One. 2025 Mar 31;20(3):e0320084. doi: 10.1371/journal.pone.0320084 (PMC11957329; doi:10.1371/journal.pone.0320084)
Supplement: S1 Appendix — (PDF) [file pone.0320084.s001.pdf]

S1 Appendix. Depression Calculation Method

This appendix presents information on the use of network data collection and natural language processing technology to calculate a depression value. First, a depression dictionary is constructed. The dictionary includes three parts: depression seed words, Weibo feature words and Chinese emotional words, including emotional words, negative words and degree adverbs. To construct basic depression words artificially via the depression discrimination scale, text rank, TF-IDF and other natural language processing methods are used to extract the feature words of depression in the text of the depression microblog. To obtain the synonyms of the above three types of words, we select the “How Net” sentiment analysis degree adverbs, data negative words, and the basic words of depression constructed in this study as the basic Chinese sentiment dictionary. After constructing the dictionary is constructed, the word segmentation tool is used to segment the microblog text, and the part-of-speech tagging of the word segmentation is realized by traversing the microblog text. The depression value of the microblog text is obtained using the calculation equation of the depression value. Finally, the Baidu Brain AI open platform is used to verify the calculation results. The specific calculation method is as follows:

1. Constructing a depression emotion dictionary

(1) Construction of depression seed words on the depression scale

In 1967, American clinical psychologist A. T. Baker divided depression into three dimensions: negative attitudes, suicide and negative emotions such as pessimism and helplessness, which represent psychological characteristics; physical symptoms, such as fatigue and poor sleep; and difficulty operating, that is, feeling that work is more difficult than before, which represents behavior.

As the Beck Depression Inventory (BDI) offers the option of ‘I am too sad or unpleasant’, the study extracts ‘sadness’ as the basic word. The six depression scales, used were the BDI, the Patient Health Questionnaire (PHQ-9), the Center for Epidemiological Studies-Depression (CES-D), the Self-Rating Depression Scale (SDS), the Hamilton Depression Scale (HDS), and the Diagnostic and Statistical Manual of Mental Disorders, fifth edition (DSM-V) checklist. The study extracts keywords for each question content of each scale, including psychological characteristics, physical symptoms, and behavioral performance. When people talk about depression, they also discuss disease names and prevention and control treatments. Combined with the three dimensions of depression, a total of 132 depression seed words were included.

(2) Constructing Weibo depression feature words

Some studies have used Weibo texts as a corpus to obtain depression-related vocabulary. The main content of the sentiment analysis in this study is microblog text. For the convenience of users who share common interests or hobbies to gather together to communicate about the same topic, Weibo established a ‘super-topics’ online community function. Many netizens who suffer from depression, medical workers with similar experiences, or individuals in related fields communicate by focusing on the ‘depression’ super-topic. Users who follow the super-topic can browse all the microblog content of the topic without paying attention to the blogger who posted it. When a blog

post is posted on a related topic, the post contains the '#depression#' logo and is displayed on the topic page. Depression in microblog text contains words such as #depression and #hypertalk. Therefore, we obtained the Weibo post text under the 'depression' super-topic. This study collected 61126 depression microblog texts from related hypertalk, used the Jieba tool for word segmentation, extracted keywords through the text rank process, increased the weight of relevant keywords according to the depression dictionary, and combined the TF-IDF algorithm to extract depression-related keywords. The top 50 depression keywords are added to the dictionary as feature words.

(3) Selected Chinese sentiment dictionary

Current Chinese dictionaries in the field of natural language processing include the BosonNLP sentiment dictionary, 'How Net' sentiment analysis word set, and data negative words. The BosonNLP sentiment dictionary has been used many times for sentiment analysis. All words in the dictionary are marked with scores ranging from -7 to 7. The higher the score is, the more positive the emotion is, including many network terms. There is also high coverage of Weibo texts, such as 'Zhen Sang Xin', and 'Tubing', which can be used as a basic sentiment score dictionary for calculating depression values. Negative words use data to negate words, and degree adverbs use the "How Net" sentiment analysis word set.

(4) Construction of a dictionary for this study

This study used Python's Chinese synonym toolkit to obtain 167 synonyms of seed words and microblog feature words, some of which did not generate synonyms. Two researchers checked the results of the toolkit and discussed and deleted inappropriate synonyms, with a consistency rate of 94%. Finally, 653 basic words related to depression, including Weibo feature words, were obtained (as shown in Appendix 2).

The study's dictionary was constructed by combining the above three types of depression emotion basic words, degree adverbs, and negative words.

2. Calculation of depressive mood values

When calculating the depression value of each microblog, the word segmentation tool is used to label the word categories of each microblog text in order, mark the emotional words, negative words and degree adverbs of each text, and then traverse the text. The depression value is calculated according to the weights and scores of various words in the dictionary.

The calculation rule is as follows:

$$\mathrm{dep} = \sum_{\mathrm{i}=1}^{n} (-1)\wedge Nn \times Ag \times Dw \times Es$$

where *Dep* is the depression value of each Weibo post, *Nn* is the number of negative words, *Ag* is the degree of adverbs, *Dw* is the weight of each word in the depression dictionary, *Es* is the score of emotional words in the BosonNLP sentiment dictionary, *i* is an emotional word, and *n* is the number of all emotional words in the text. *Ag* is divided into six types of degree adverbs, namely, extreme/most, super, very, relatively, slight, and owe, and assigned values of 2, 1.75, 1.5,

1, 0.5 and 0.25, respectively. According to the core features of major depressive disorder, the *Dw* calculation assigns weights ranging from 1 to 7 to different vocabulary categories in the constructed depression emotion dictionary.

For the convenience of observation, when the end of the running calculation is reached, the dep results are multiplied by (-1), using a sigmoid function to achieve 0-1 normalization, and then multiplied by 10; the greater the value, the greater the level of depressive tendency. The total depression tendency value must be added to the depression values of all Weibo posts in that city on that day.

Definition rules of various vocabulary weights in dictionaries:

Definitions: *Evalue* = the depression score of words in the dictionary of depression in this study (represented by 'Dictionary A'), *Bs* = the emotion score of words in the BosonNLP sentiment dictionary (represented by 'Dictionary B'), and Dw = the weight of emotional words in the dictionary of this study.

Title: Known Dictionary A contains Dictionary B, $Evalue = Bs * Dw$; the absolute value of the emotional score of words in the BosonNLP sentiment dictionary ranges from 0 to 7, that is, $0 < | Bs | < 7$, Bs. Max represents the word with the highest emotional score in Dictionary B; and the maximum absolute value of the word is -3.656241866 (nightmare). The closer the value is to 0, the more neutral the word's emotion is.

Finding: The emotional score of words expressing depression in Dictionary A is greater than that of other emotional words; that is, after the weight is multiplied, *Bs* is greater than Bs.max. Thus, how should we assign *Dw*?

To implement $Evalue > | Bs.\max |$

It is known that $0 < | Bs.\max | < 7$

So $Evalue > 7 > | Bs.\max |$

That is, $Bs * Dw > 7 > | Bs.\max |$

When *Dw* is assigned a maximum of 7, the value is greater than that of *Bs*.max

The core features are assigned the highest weight; that is, psychological characteristics are assigned a weight of 7.

For other behavioral characteristics and somatic symptoms, the value is smaller; thus, the weight assigned is 6.

Somatic symptoms involve other non-depressive physiological problems; thus, the assignment is smaller than that of the behavioral characteristics, which are assigned a weight of 5.

Prevention and control treatments suggest that the possibility of depression is greater but may also involve more popular science articles; thus, they are assigned a weight of 5.5.

The Weibo feature words are assigned a weight of 3.

The remaining words are assigned a weight of 1 using the original BosonNLP dictionary assignment.
