# Supplementary material for: The impact of fine particulate matter on depression: Evidence from social media in China
Source: PLoS One. 2025 Mar 31;20(3):e0320084. doi: 10.1371/journal.pone.0320084 (PMC11957329; doi:10.1371/journal.pone.0320084)
Supplement: S2 Appendix — (PDF) [file pone.0320084.s002.pdf]

S2 Appendix. Depression Basic Words

S1 Table. Basic Words of Depression

Existing words	Category	Synonyms and associated words
depression	psychological	obsessive-compulsive disorder, schizophrenia, manic depression
pessimism	psychological	weak, worried, bearish
sadness	psychological	grief, sentimentality, heartache, despair, regret
anxiety	psychological	impatience, fear, panic, resentment, worry, disgust
dissatisfaction	psychological	strong dissatisfaction, indignation, blame, anger, displeasure
heavy	psychological	vulnerability, misery, depth, weakness, helplessness, burden
low	psychological	poor, unstable, extremely poor, plummeting, depressed, excited
hostility	psychological	caution, hatred, prejudice, resentment, hostility, suspicion, hatred, frustration, prejudice
annoyance	psychological	distress, confusion, illness, sorrow, pain, suffering
fidgety	psychological	tiredness, boredom, drowsiness, upset, irritability
alone	psychological	lonely, solitude, sadness, anxiety
fear	psychological	dread, worry, disgust, hatred, shame, cowardice
fantasy	psychological	dream, illusion, imagination, nothingness
anxious	psychological	insecurity, inferiority, confusion, irritability
terrified	psychological	stunned, embarrassed, frightened, at a loss, panic
depressed	psychological	disappointment, sadness, regret, guilt, embarrassment, silent, indecisive, anxious, reluctant to part, good temper
despair	psychological	grievance and heartbreak
subdued	psychological	difficulty, frustration, hesitation
sad	psychological	distressed, uncomfortable, ashamed
guilt	psychological	guilt, remorse, regret
be down in spirits	psychological	asthma, upset, syncope, weakness, pain, nervousness
fail	psychological	failure, near success, frustration, futility
sigh	psychological	sigh, lament, pity, melancholy
hate	psychological	fear, despise, disdain
pain	psychological	suffering
shrink back	psychological	retreat, yield, be discouraged, drift with the current, and face a dilemma
delusion	psychological	ignorance, narcissism, nihility, morbid state, paranoia, obsession, crankiness
hopeless	psychological	unable to turn back, desperate, eager, determined, disheartened, settled, gloomy, unfinished
useless	psychological	no value, just, divorced from reality, inappropriate
sorrow	psychological	sadness
worried	psychological	doubt, panic, concern
guilty	psychological	confessions, felonies, capital offences
inferiority	psychological	inferiority complex, neuroticism, sentimentality, arrogance

self-knowledge	psychological	be utterly helpless, for fear that, afraid, see also
silent	behavior	meditation, confusion, madness, decadence
slow	behavior	sluggish, weak, too slow, sluggish, quick, lack of stamina
effort	behavior	care and effort
hallucination	behavior	illusion, fear, dream, guilt, sickness, hysteria
intense	behavior	high pitched, loud, solemn and stirring
cry	behavior	cry loudly, bitterly, shed tears, cried, broke down in tears, burst into tears
weep	behavior	shouting, groaning, trembling, wailing
be out of spirits	behavior	be absent-minded
hesitant	behavior	vacillate, sullen, discontented
self-mutilation	behavior	self-injury, homicide, sexual abuse, mental disorder, abuse
commit suicide	behavior	jumping from a building, committing suicide, being killed, self-immolation, hanging oneself, committing suicide, taking poison, attempting suicide
be on tenterhooks	behavior	agonizing
be unable to eat any more	body	spitting blood, hunger, nausea, weakness
nightmare	body	incubus, catastrophe, misfortune, despair
weakness	body	sluggish, gloomy, difficult and weak
indigestion	body	abdominal distension, gastritis, edema, gastric ulcer, stomachache, asthma, hemorrhoids, hematochezia
tired	body	numbness, fatigue, dizziness, hunger and thirst, exhaustion
fatigue	body	insufficient sleep, soreness, dyspnea, dizziness
insomnia	body	depression, palpitation, dizziness, neurasthenia, tinnitus, fever
poor appetite	body	abdominal pain, chills
palpitation	body	chest pain, syncope
pain	body	convulsions
lose weight	body	gain weight
drug	prevent & control	tablets, antibiotics, pharmaceuticals, preparations, pills, painkillers, western medicine
side effects	prevent & control	complications, allergic reactions, adverse reactions, side effects, symptoms, drug resistance
mental illness	prevent & control	mental disorders, mental hospital, epilepsy, delusions, mental patients
psychiatry	prevent & control	physician, specialist, obstetrics and gynecology, neurosurgery, obstetrics, nursing
psychological consultation	prevent & control	counseling, consultant, psychologist, mental health, medical, social work
scales	prevent & control	tests, subjects
treatment	prevent & control	psychotherapy, diagnosis and treatment, medication, clinic, outpatient, hospital
live	Weibo	starve, die, living is worse than dying
no	Weibo	do not dare, refuse, should not, cannot, cannot bear, have no courage
hope	Weibo	expect, want, believe, take this opportunity
like	Weibo	loving, liking, preferring, watching, favorite, playing
happy	Weibo	excited, full of joy and pride
doctor	Weibo	physicians, psychologists, psychiatrists, nurses
patient	Weibo	subject

life	Weibo	mind, soul, body, faith, afterlife
uncomfortable	Weibo	discomfort and holding back
body	Weibo	soma
leave	Weibo	leave, escape, move out
spirit	Weibo	thought
psychology	Weibo	interpersonal relationship, mind, mentality, personality, self-esteem, morbid
take medicine	Weibo	fever
interest	Weibo	attractive force
symptom	Weibo	disease
heart	Weibo	inner world, heart bottom, state of mind, thoughts, sensibility and depth
collapse	Weibo	disintegrate, disintegration, destruction, wither away, fall apart, doom
control	Weibo	adjust, dominate, control and suppress
severe	Weibo	moderate, mild, chronic, migraine, systemic
sleep	Weibo	get up, take a bath, fall asleep, go to bed
give up	Weibo	exit, abandon, choose to exit
face	Weibo	face up to, fear nothing, face reality, know well, let go
friend	Weibo	friends, colleagues, relatives, classmates, girlfriends, boyfriends, old friends, neighbors
life	Weibo	mood
time	Weibo	waiting time, interval, distance, time delay, microseconds, rest time
look	Weibo	think
become	Weibo	seem
understand	Weibo	understand
be willing	Weibo	willing, planning, eager, willing to do
accept	Weibo	admit

**Category**	**Words without synonyms**
psychological	punishment, worry about the body, guilt, too much attention to the body, meaningless, meaningless, no hope, no pleasure, low mood, lack of interest, loss of interest, threatened, hate yourself, the mind is not clear, no sense of value, like the morning, want to cry, distorted image, excited, sick, self-incrimination
behavior	inhibited activities, no vitality, difficult to concentrate, difficult to focus, difficult to continue life, restless mind, accelerated heartbeat, hypochondria, unpleasant communication with people, difficult to do things
body	headache, drowsiness, eating too much, menstrual disorders, fatigue, lack of energy, difficulty in falling asleep, loss of appetite, loss of appetite, unstable sleep, poor sleep, shallow sleep, too much sleep, lack of strength, weight loss, poor appetite, loss of libido, early awakening, decreased attention, constipation
prevent & control	escitalopram, paroxetine, citalopram, Escitalopram Oxalate Tablets, Venlafaxine, Duloxetine, mental health center, psychology, electroconvulsive, sertraline, fluoxetine
Weibo	world, energy, feeling

Note: English is different from Chinese. Some Chinese words have no synonyms, whereas English words may have synonyms and vice versa. Thus, the English version of the glossary is either lacking or supplemented.
