# Supplementary material for: The impact of fine particulate matter on depression: Evidence from social media in China
Source: PLoS One. 2025 Mar 31;20(3):e0320084. doi: 10.1371/journal.pone.0320084 (PMC11957329; doi:10.1371/journal.pone.0320084)
Supplement: S3 Appendix — (PDF) [file pone.0320084.s003.pdf]

S3 Appendix. Method for calculating instrumental variables

The instrumental variable is the spillover effect of air pollution in the headwind area, that is, the weighted value of pollutants in the surrounding city, which is found using the weighted sum of PM2.5 concentrations in the adjacent area for the day as the instrumental variable ($\mathrm{Neighbour}_{it}$). The calculation formula (1) is as follows:

$$Neighbour_{it} = \sum_j \left(w_{ijt} \times pe_{jt} \big/ d_{ij}\right),\ 100km < d_{ij} < 200km \quad (1)$$

where pe_{jt} is the PM2.5 concentration of city *j* on day *t*, available from official data; d_{ij} is the distance between local city *i* and city *j*, and the selection range is between 100 km and 200 km. The calculation method is based on the Baidu map API and is used to obtain the longitude and latitude of each city. Python is subsequently used to calculate the distance between two cities according to the longitude and latitude. The calculation formula is as follows:

$$\mathrm{haversin}\left(\frac{\mathrm{d_{ij}}}{\mathrm{R}}\right) = \mathrm{haversin}(\varphi_j - \varphi_i) + \cos(\varphi_i)\cos(\varphi_j)\mathrm{haversin}(\Delta\lambda) \quad (2)$$

where *R* is the radius of the Earth, averaged at 6371 K; φ_j and φ_i denote the latitudes of the two cities; and $\Delta\lambda$ represents the difference between the two latitudes.

$$\mathrm{haversin}(\theta) = (1 - \cos(\theta))/2,\ \theta \text{ can be replaced by } \varphi_j - \varphi_i \text{ or } \frac{\mathrm{d_{ij}}}{\mathrm{R}} \quad (3)$$

The weight w_{ijt} is distributed according to the relative direction of city *j* and city *i* and the wind direction of city *i* on day *t*. If city *j* is at the upper outlet, then the distribution right is significant. The specific calculation is shown in Formula (4):

$$\begin{cases} w_{ijt} = \cos\left(\theta_{\mathrm{Wit}} - \theta_{Dij}\right) & w_{ijt} \geq 0 \\ w_{ijt} = 0 & w_{ijt} < 0 \end{cases} \quad (4)$$

where θ_{Wit} is the wind direction angle of city i on day t, which is converted as follows:

S2 Table. Angles corresponding to different directions

Direction	Angle
North	0°
North by northeast	22.5°
Northeast	45°
East-northeast	67.5°
East	90°
East by southeast	112.5°
Southeast	135°
South by southeast	157.5°
South	180°
South by southwest	202.5°
Southwest	225°
West by southwest	247.5°

West	270°
West by northwest	292.5°
Northwest	315°
North-northwest	337.5°

where θ_{Dij} is the azimuth of city *j* relative to city *i*, which is calculated as described below.

We need to determine the latitude and longitude of two cities, namely, city i (*Ig, Iw*) and city j (*Jg, Jw*):

$$\theta_{\mathrm{Dij}} = \arctan((\mathrm{Jg-Ig}) * \mathrm{Cos}(\mathrm{Jw})/(\mathrm{Jw-Iw})) \qquad (5)$$

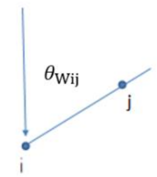

S1 Fig. θ_{Wij} Schematic diagram

In summary, the specific algorithm of the instrumental variable is as follows:

(1) Select city *j*;

(2) Calculate θ_{Wit} and θ_{Dij};

(3) Calculate the weight *w* using Formula (3);

Note: If *w* is less than 0, then take 0;

(4) Formula (1) is the weighted sum of the pollutant emissions of the surrounding cities on the same day.
