# Supplementary material for: The impact of fine particulate matter on depression: Evidence from social media in China
Source: PLoS One. 2025 Mar 31;20(3):e0320084. doi: 10.1371/journal.pone.0320084 (PMC11957329; doi:10.1371/journal.pone.0320084)
Supplement: S4 Appendix — (PDF) [file pone.0320084.s004.pdf]

S4 Appendix. Monetization of Mental Health Benefits

1. Method of calculation

According to the results of this study, for every 1 μg/m^3 increase in PM2.5 due to air pollution, the likelihood of depression among residents increases by 0.0559%. To determine the medical cost related to depression caused by air pollution, we need to know the medical cost of each patient with depression. Following existing research, the calculation method is shown in the following formula:

$$PdeCost = DeCost \times \beta_1 \times N \times People \qquad (6)$$

where *PdeCost* represents the medical cost for depression caused by air pollution (unit: $), *DeCost* represents the medical cost for each depression patient, and β_1 represents the probability (%) of each resident experiencing depression due to an increase of 1 μg/m^3 in the air pollutant PM2.5. In our study, the possibility of depression β_1 was 0.0559%, where *N* represents the multiple increases in the concentration of the air pollutant PM2.5 (default value is 1, no unit), and *People* represents the number of residents (default value is 1 resident).

2. Results of this study on medical costs

According to the literature, the value range of *Decost* ranges from $300 to $2500. Combining the formula, *N* and *People* have different values based on different situations, as shown in the table below. $PdeCost_{min}$ represents the minimum medical cost for depression caused by air pollution when *Decost* is $300, and $PdeCost_{max}$ represents the maximum medical cost for depression caused by air pollution when *Decost* is $2500.

(1) When *N* is 1 and *People* is 1, that is, when the air pollution concentration increases by 1 μg/m^3, each resident may have to bear the potential medical cost for depression; then, $PdeCost_{min}$= 300×0.0559%.×1×1= 0.17, and $PdeCost_{max}$= 2500×0.0559%×1×1= 1.4. Therefore, the value of *PdeCost* ranges from $0.17 to $1.4, which means that the medical cost for depression caused by air pollution ranges from $0.17 to $1.4 for each resident.

(2) The sample of this study is Weibo users represented by Chinese netizens, yet not all age groups of Chinese people use Weibo. Therefore, for the age distributions of Weibo users and depression patients investigated in previous studies, see S4 Table. The sample of this study can estimate that the proportion of depression patients is about 45% to 86%. According to the China Mental Health Survey, while 95 million people in China suffer from depression, only 8.2% of depressed patients in China actively seek medical treatment. Thus, the value range of *People* is from 3.51 million (multiply 95 million by 45% and then by 8.2%) to 6.7million (multiply 95 million by 86% and then by 8.2%), and the values of the other variables remain unchanged. According to Formula (6), $PdeCost_{\mathrm{min}}$ is $0.59 million, and $PdeCost_{\mathrm{max}}$ is $9.36 million. This shows that a nationwide 1 μg/m^3 reduction in particulate matter concentration would generate nearly $0.59 million to $9.36 million annually in economic benefits.

(3) From 2016 to 2019, the average annual concentration of PM2.5 in China decreased by 8.45 μg/m^3. According to Formula (6), *N*=8.45/1=8.45, and the values of the other variables

remain unchanged. Thus, $PdeCost_{min}$ is \$1.42, and $PdeCost_{max}$ is \$11.81. Therefore, during this period, due to the increase in air pollution, the average annual per capita inpatient costs decreased by \$1.42 to \$11.81.

(4) If we calculate the average annual national depression medical expenses that have been reduced due to improved air quality over the past six years, *People* ranges from 3.51 million to 6.31 million, N=8.45 and the values of the other variables remain unchanged. According to Formula (6), from 2016 to 2019, the average annual cost range for reducing medical expenses for depression nationwide due to improved air quality ranged from \$4.97 million to \$79.12 million in China, indicating that air pollution results in significant economic expenditures.

S3 Table. Four medical costs situations in this study

DeCost (US\$)	β_1	***N***	***People***	$PdeCost_{min}$ (US\$)	$PdeCost_{max}$ (US\$)
300 to 2500	0.0559 %	1	1	0.17 (300 ×0.0559%×1×1)	1.4 (2500 ×0.0559%×1×1)
		1	3.51 million to 6.7 million	0.59 million (300 ×0.0559%×1×3.51 million)	9.36 million (2500 ×0.0559%×1×6.7 million)
		8.45	1	1.42 (300 ×0.0559%×8.45×1)	11.81 (2500 ×0.0559%×8.45×1)
		8.45	3.51 million to 6.7 million	4.97 million (300 ×0.0559%×8.45×3.51 million)	79.12 million (2500 ×0.0559%×8.45×6.7 million)

S4 Table. Age composition of depression patients and Weibo users

	**Depression patients**			**Weibo users**
	Chinese Mental Health Investigation Team	Shanghai medical data from 2016 to 2019	National Mental Health Database	
⩽18	-	0.86%	**25.23%**	11.63%
19-59	78.25%	43.45%	73.26%	87.37%
>60	21.75%	55.69%	1.51%	1%

Note: Weibo users represent a maximum of 11.63% of teenagers younger than 18, which is 13.6% less than the maximum proportion of 25.23%. Therefore, the estimated proportion that meets the criteria is approximately 86.4% (equal to 1 minus 13.6%). Weibo users represent a maximum of 1% of the elderly population aged 60 and above, which is 54.69% lower than the maximum of 55.69%. At this point, the estimated proportion is approximately 45.31% (equal to 1 minus 54.69%). Therefore, as a representative of Chinese netizens, Weibo can estimate that the proportion of depression patients ranges from approximately 45% to 86%.
