# Supplementary material for: The impact of fine particulate matter on depression: Evidence from social media in China
Source: PLoS One. 2025 Mar 31;20(3):e0320084. doi: 10.1371/journal.pone.0320084 (PMC11957329; doi:10.1371/journal.pone.0320084)
Supplement: S5 Appendix — (PDF) [file pone.0320084.s005.pdf]

S5 Appendix. 284 prefecture-level cities and their respective provinces

S5 Table. 284 prefecture-level cities and their respective provinces

Number	Province	City	Number	Province	City
1	Anhui	Anqing	144	Jilin	Baishan
2	Anhui	Bengbu	145	Jilin	Jilin
3	Anhui	Bozhou	146	Jilin	Liaoyuan
4	Anhui	Chizhou	147	Jilin	Siping
5	Anhui	Chuzhou	148	Jilin	Songyuan
6	Anhui	Fuyang	149	Jilin	Tonghua
7	Anhui	Hefei	150	Jilin	Changchun
8	Anhui	Huainan	151	Jiangsu	Changzhou
9	Anhui	Huangshan	152	Jiangsu	Huaian
10	Anhui	Lu'an	153	Jiangsu	Lianyungang
11	Anhui	Ma'anshan	154	Jiangsu	Nanjing
12	Anhui	Suzhou	155	Jiangsu	Nantong
13	Anhui	Tongling	156	Jiangsu	Suzhou
14	Anhui	Wuhu	157	Jiangsu	Suqian
15	Beijing Shi	Beijing	158	Jiangsu	Taizhou
16	Chongqing Shi	Chongqing	159	Jiangsu	Wuxi
17	Fujian	Fuzhou	160	Jiangsu	Xuzhou
18	Fujian	Longyan	161	Jiangsu	Yancheng
19	Fujian	Nanping	162	Jiangsu	Yangzhou
20	Fujian	Putian	163	Jiangsu	Zhenjiang
21	Fujian	Quanzhou	164	Jiangxi	Ganzhou
22	Fujian	Sanming	165	Jiangxi	Ji'an
23	Fujian	Xiamen	166	Jiangxi	Jiujiang
24	Fujian	Zhangzhou	167	Jiangxi	Nanchang
25	Gansu	Baiyin	168	Jiangxi	Pingxiang
26	Gansu	Dingxi	169	Jiangxi	Shangrao
27	Gansu	Jiayuguan	170	Jiangxi	Yichun
28	Gansu	Jinchang	171	Jiangxi	Yingtan
29	Gansu	Jiuquan	172	Liaoning	Anshan
30	Gansu	Lanzhou	173	Liaoning	Benxi
31	Gansu	Linxia	174	Liaoning	Chaoyang
32	Gansu	Pingliang	175	Liaoning	Dalian
33	Gansu	Qingyang	176	Liaoning	Dandong
34	Gansu	Tianshui	177	Liaoning	Fushun
35	Gansu	Wuwei	178	Liaoning	Fuxin
36	Gansu	Zhangye	179	Liaoning	Huludao
37	Guangdong	Chaozhou	180	Liaoning	Jinzhou
38	Guangdong	Dongguan	181	Liaoning	Liaoyang
39	Guangdong	Foshan	182	Liaoning	Panjin
40	Guangdong	Guangzhou	183	Liaoning	Shenyang

41	Guangdong	Heyuan	184	Liaoning	Yingkou
42	Guangdong	Huizhou	185	Nei Mongol Zizhiqu	Baotou
43	Guangdong	Jiangmen	186	Nei Mongol Zizhiqu	Chifeng
44	Guangdong	Jieyang	187	Nei Mongol Zizhiqu	Ordos
45	Guangdong	Maoming	188	Nei Mongol Zizhiqu	Hohhot
46	Guangdong	Meizhou	189	Nei Mongol Zizhiqu	Tongliao
47	Guangdong	Qingyuan	190	Nei Mongol Zizhiqu	Wuhai
48	Guangdong	Shantou	191	Ningxia Huizu Zizhiqu	Guyuan
49	Guangdong	Shanwei	192	Ningxia Huizu Zizhiqu	Shizuishan
50	Guangdong	Shaoguan	193	Ningxia Huizu Zizhiqu	Wuzhong
51	Guangdong	Shenzhen	194	Ningxia Huizu Zizhiqu	Yinchuan
52	Guangdong	Yangjiang	195	Ningxia Huizu Zizhiqu	Zhongwei
53	Guangdong	Yunfu	196	Qinghai	Guoluo
54	Guangdong	Zhanjiang	197	Qinghai	Haibei
55	Guangdong	Zhaoqing	198	Qinghai	Hainan
56	Guangdong	Zhongshan	199	Qinghai	Haixi
57	Guangdong	Zhuhai	200	Qinghai	Huangnan
58	Guangxi Zhuangzu Zizhiqu	Baise	201	Qinghai	Xining
59	Guangxi Zhuangzu Zizhiqu	Beihai	202	Qinghai	Yushu
60	Guangxi Zhuangzu Zizhiqu	Chongzuo	203	Shandong	Binzhou
61	Guangxi Zhuangzu Zizhiqu	Fangchenggang	204	Shandong	Dezhou
62	Guangxi Zhuangzu Zizhiqu	Guigang	205	Shandong	Dongying
63	Guangxi Zhuangzu Zizhiqu	Guilin	206	Shandong	Heze
64	Guangxi Zhuangzu Zizhiqu	Hechi	207	Shandong	Jinan
65	Guangxi Zhuangzu Zizhiqu	Hezhou	208	Shandong	Jining
66	Guangxi Zhuangzu Zizhiqu	Laibin	209	Shandong	Linyi
67	Guangxi Zhuangzu Zizhiqu	Liuzhou	210	Shandong	Qingdao
68	Guangxi Zhuangzu Zizhiqu	Nanning	211	Shandong	Rizhao
69	Guangxi Zhuangzu Zizhiqu	Qinzhou	212	Shandong	Taian
70	Guangxi Zhuangzu Zizhiqu	Wuzhou	213	Shandong	Weihai
71	Guangxi Zhuangzu Zizhiqu	Yulin	214	Shandong	Weifang
72	Guizhou	Anshun	215	Shandong	Yantai
73	Guizhou	Bijie	216	Shandong	Zaozhuang
74	Guizhou	Guiyang	217	Shandong	Zibo
75	Guizhou	Tongren	218	Shanxi	Datong
76	Guizhou	Zunyi	219	Shanxi	Jincheng
77	Hainan	Haikou	220	Shanxi	Jinzhong
78	Hainan	Sanya	221	Shanxi	Linfen
79	Hebei	Baoding	222	Shanxi	Lvliang
80	Hebei	Cangzhou	223	Shanxi	Shuozhou
81	Hebei	Chengde	224	Shanxi	Taiyuan
82	Hebei	Handan	225	Shanxi	Xinzhou
83	Hebei	Hengshui	226	Shanxi	Yangquan

84	Hebei	Langfang	227	Shanxi	Yuncheng
85	Hebei	Qinhuangdao	228	Shanxi	Changzhi
86	Hebei	Shijiazhuang	229	Shaanxi	Baoji
87	Hebei	Tangshan	230	Shaanxi	Hanzhong
88	Hebei	Xingtai	231	Shaanxi	Shangluo
89	Hebei	Zhangjiakou	232	Shaanxi	Tongchuan
90	Henan	Anyang	233	Shaanxi	Weinan
91	Henan	Hebi	234	Shaanxi	Xi'an
92	Henan	Jiaozuo	235	Shaanxi	Xianyang
93	Henan	Kaifeng	236	Shaanxi	Yanan
94	Henan	Luoyang	237	Shaanxi	Yulin
95	Henan	Leihe	238	Shanghai Shi	Shanghai
96	Henan	Nanyang	239	Sichuan	Aba
97	Henan	Pingdingshan	240	Sichuan	Bazhong
98	Henan	Puyang	241	Sichuan	Chengdong
99	Henan	Sanmenxia	242	Sichuan	Dazhou
100	Henan	Shangqiu	243	Sichuan	Deyang
101	Henan	Xinxiang	244	Sichuan	Ganzi
102	Henan	Xinyang	245	Sichuan	Guangan
103	Henan	Xuchang	246	Sichuan	Guangyuan
104	Henan	Zhengzhou	247	Sichuan	Leshan
105	Henan	Zhoukou	248	Sichuan	Liangshan
106	Henan	Zhumadian	249	Sichuan	Luzhou
107	Heilongjiang	Daqing	250	Sichuan	Meishan
108	Heilongjiang	Da Hinggan Ling	251	Sichuan	Mianyang
109	Heilongjiang	Harbin	252	Sichuan	Nanchong
110	Heilongjiang	Hegang	253	Sichuan	Neijiang
111	Heilongjiang	Heihe	254	Sichuan	Panzhihua
112	Heilongjiang	Jixi	255	Sichuan	Suining
113	Heilongjiang	Jiamusi	256	Sichuan	Ya'an
114	Heilongjiang	Mudanjiang	257	Sichuan	Yibin
115	Heilongjiang	Qitaihe	258	Sichuan	Ziyang
116	Heilongjiang	Qiqihaer	259	Sichuan	Zigong
117	Heilongjiang	Shuangyashan	260	Tianjin Shi	Tianjin
118	Heilongjiang	Suihua	261	Yunnan	Baoshan
119	Heilongjiang	Yichun	262	Yunnan	Chuxiong
120	Hubei	Ezhou	263	Yunnan	Dali
121	Hubei	Huanggang	264	Yunnan	Dehong
122	Hubei	Huangshi	265	Yunnan	Honghe
123	Hubei	Jingmen	266	Yunnan	Kunming
124	Hubei	Jingzhou	267	Yunnan	Lijiang
125	Hubei	Shiyan	268	Yunnan	Lincang
126	Hubei	Suizhou	269	Yunnan	Nujiang

127	Hubei	Wuhan	270	Yunnan	Qujing
128	Hubei	Xianning	271	Yunnan	Wenshan
129	Hubei	Xiangyang	272	Yunnan	Yuxi
130	Hubei	Xiaogan	273	Yunnan	Zhaotong
131	Hubei	Yichang	274	Zhejiang	Hangzhou
132	Hunan	Changde	275	Zhejiang	Huzhou
133	Hunan	Chenzhou	276	Zhejiang	Jiaxing
134	Hunan	Huaihua	277	Zhejiang	Jinhua
135	Hunan	Loudi	278	Zhejiang	Lishui
136	Hunan	Xiangtan	279	Zhejiang	Ningbo
137	Hunan	Yiyang	280	Zhejiang	Quzhou
138	Hunan	Yongzhou	281	Zhejiang	Shaoxing
139	Hunan	Yueyang	282	Zhejiang	Taizhou
140	Hunan	Zhangjiajie	283	Zhejiang	Wenzhou
141	Hunan	Changsha	284	Zhejiang	Zhoushan
142	Hunan	Zhuzhou			
143	Jilin	Baicheng			

3 Note: Sorted by the first letter of the phonetic alphabet of the province where the city is located.
